# Autoimmune Hypophysitis With Systemic Lupus Erythematosus: A Case Report and Literature Review

**DOI:** 10.3389/fendo.2020.579436

**Published:** 2020-10-07

**Authors:** Pengyue Xiang, Qiuxia Wu, Hua Zhang, Chaoyang Luo, Huajie Zou

**Affiliations:** ^1^ Department of Endocrinology, Jingmen No.2 People’s Hospital, Jingmen, China; ^2^ Department of Endocrinology, Tongji Hospital, Tongji Medical College, Huazhong University of Science and Technology, Wuhan, China

**Keywords:** autoimmune hypophysitis, systemic lupus erythematosus, anterior pituitary hypofunction, autoimmune disease, sella turcica

## Abstract

**Background:**

Autoimmune hypophysitis (AH) is a primary autoimmune inflammatory disorder of the pituitary gland, which usually presents as a mass in the sella turcica. Systemic lupus erythematosus (SLE) is another inflammatory disorder in which the immune system attacks healthy cells and tissues throughout the body. Although both diseases are autoimmune disorders, they rarely coexist, and the relationship between them is unclear.

**Case Report:**

A 66-year-old man was evaluated at the endocrinology clinic because of worsening fatigue, anorexia, drowsiness, and leg oedema. Examination revealed alertness impairment and lower limb oedema. Laboratory tests showed anterior pituitary hypofunction. The treatment approach, with glucocorticoids and immunosuppressive agents, resulted in long-term remission of symptoms of hypopituitarism and hyponatraemia.

**Conclusions:**

Our case demonstrates a potential association between AH and SLE. AH may need to be considered in the evaluation of SLE patients with headache, hyperprolactinemia, a pituitary mass, and hypopituitarism.

## Introduction

Autoimmune hypophysitis (AH), also known as lymphocytic hypophysitis, is a rare inflammatory disorder that can affect the anterior, posterior or both pituitary lobes (1). Most cases have been diagnosed in women during late pregnancy or the postpartum period (2). Systemic lupus erythematosus (SLE) is a condition where the immune system attacks healthy cells as well as tissues around the body (3). AH is rarely associated with rheumatic diseases, and only 1.3% of AH cases have previously been described to be associated with SLE (4). Here, we describe a case of AH and SLE in a 66-year-old man presenting with fatigue, anorexia, drowsiness, and leg oedema, and we summarize all case reports of AH with SLE to provide some evidence of the pathogenesis, diagnosis and treatments.

## Case Presentation

A 66-year-old man with a history of diabetes and percutaneous transluminal coronary intervention (PCI) was evaluated at the endocrinology clinic because of worsening fatigue, anorexia, drowsiness, and leg oedema.

The patient had been followed up by one of us (P X) for 5 years because of non-insulin-dependent diabetes mellitus (NIDDM). Three years ago, the patient was diagnosed with cardiovascular disease (CVD) and treated with percutaneous coronary intervention (PCI) in the Department of Cardiology. Five months before presentation, the patient was readmitted to the Department of Cardiology because of fatigue, anorexia, drowsiness, and leg oedema. His temperature was 36.5°C, heart rate was 72 beats/min, and blood pressure was 135/85 mmHg. At the Department of Cardiology, the white-cell and differential counts and blood levels of erythrocyte sedimentation rate (ESR), high-sensitivity C-reactive protein, thyroid stimulating hormone (TSH), and free T3 (FT3) were normal; the level of free T4 (FT4) was decreased; and levels of glycated haemoglobin A1c (HbA1c), triglycerides, and brain natriuretic peptide (BNP) were elevated. The blood sodium level was 131.7 mmol/L [normal range (NR), 137 to 147]. During hospitalization, he received treatment with laxatives due to constipation, and his blood sodium level decreased to 110.3 mmol/L. Therefore, treatment with 3% NaCl was initiated, with obvious relief in symptoms. The patient was discharged when the sodium level reached 130.1 mmol/L.

**Figure 1:**
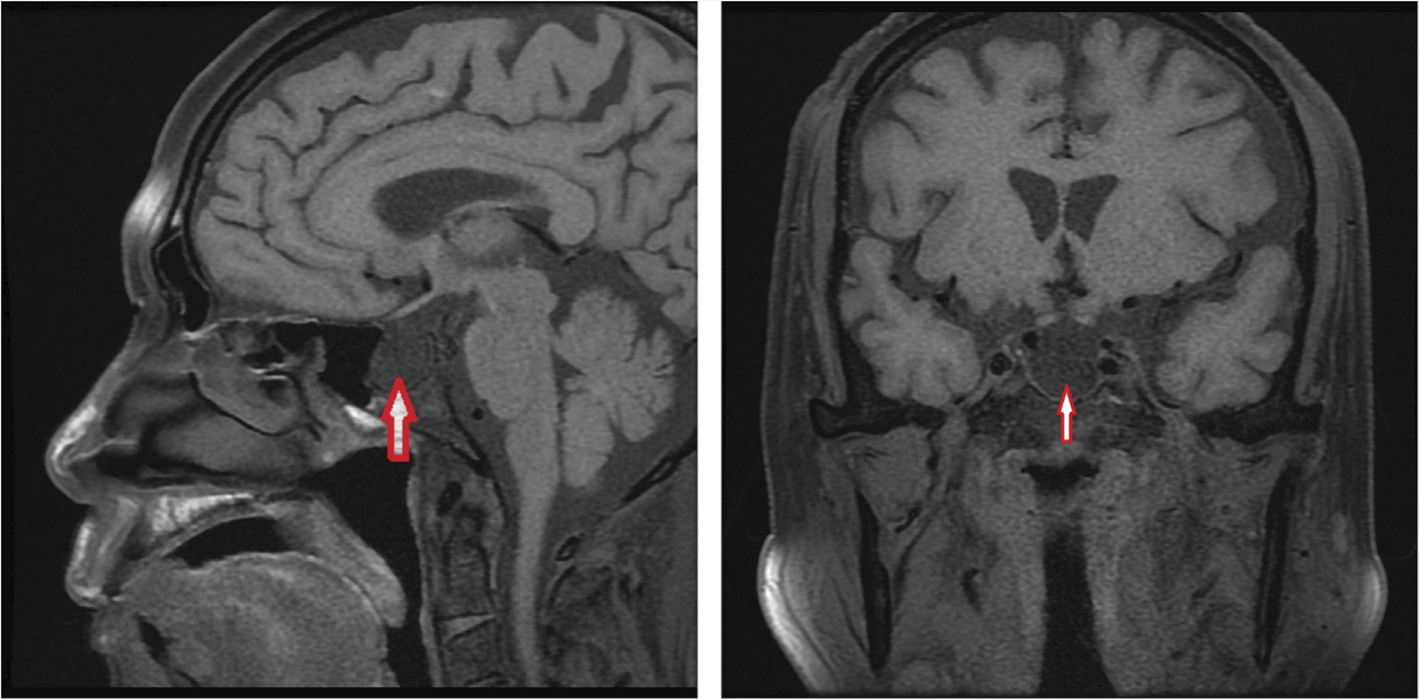
Brain MRI.

At the last visit in the endocrinology clinic, the patient complained that these symptoms had worsened during the past week. He reported no headache, dizziness, polydipsia, polyuria, or vomiting. Examination revealed alertness impairment and lower limb oedema. No trochlear nerve palsy, oculomotor nerve paralysis, or visual field defects were detected during examination. As shown in **Table 1**, laboratory tests showed anterior pituitary hypofunction. The FT3 (1.93 pg/ml; NR, 2.27 to 4.22) and FT4 (0.63 ng/dl; NR, 0.9 to 1.76) decreased without a corresponding increase in TSH (3.00 uIU/ml; NR, 0.55 to 4.78), suggesting central hypothyroidism. Follicle-stimulating hormone (FSH), luteinizing hormone (LH), and testosterone levels were all below the sensitivity of the assay, consistent with hypogonadotropic hypogonadism. Laboratory examination also showed low sodium (124 mmol/L; NR, 137 to 147) and chloride (89 mmol/L; NR, 99 to 110) and low morning cortisol (0.49 µg/dl; NR, 4.26 to 24.85) without a corresponding increase in corticotrophin (ACTH 8.14 pg/ml, NR 7.2–63.4), suggesting central hypercortisolism. The insulin-like growth factor 1 (IGF-1) level was decreased (53 ng/ml; NR, 69-211), and the serum growth hormone (GH) level was normal. Interestingly, prolactin (PRL) was slightly elevated (26.6 ng/ml; NR, 3.6 to 16.3), which occurs in approximately 18% of AH patients (5) and can decrease LH and FSH levels. Test results for rheumatoid factor (RF), antinuclear antibody (ANA), anti-Sm antibody, anti-Sjögren’s syndrome B (anti-SSB), anti-double-stranded DNA antibodies (anti-dsDNA), anti-nRNP/Sm, and anticardiolipin antibody-IgG (ACL-IgG) were positive. Test results for other relevant antibodies were negative (**Table 1**). The levels of all tumour markers were normal. The magnetic resonance imaging (MRI) scan of the pituitary gland revealed an empty sella (**Figure 1**). No abnormality was observed in the adrenal MRI.

**Table 1:** Laboratory findings.

Variable	Results	Reference values
Blood routine		
Hemoglobin (g/L)	115	130–175
Hematocrit (%)	0.320	0.4–0.5
White‑cell count (*10^9/L)	4.18	3.5–9.5
Platelet count (*10^9/L)	253	125–350
Electrolyte		
Sodium (mmol/L)	124	137–147
Potassium (mmol/L)	3.4	3.5–5.3
Chloride (mmol/L)	89.0	99–100
Calcium (mmol/L)	2.22	2.1–2.9
Blood biochemical		
Urea nitrogen (mmol/L)	2.51	3.6–9.5
Creatinine (µmol/L)	54.7	57–111
Glucose (mmol/L)	6.32	4.11–5.89
HbA1c (%)	6.80	4.27–6.07
Total protein (g/L)	67.1	65–85
Albumin (g/L)	40.9	40–55
cTnI (ng/ml)	<0.02	0–0.06
Endocrine hormones		
FT4 (ng/dl)	0.63	0.9–1.76
FT3 (pg/ml)	1.93	2.27–4.22
TSH (uIU/ml)	3.00	0.55–4.78
GH (μg/L)	0.04	≤2.47
IGF-1 (ng/ml)	53	69–211
ACTH (μg/ml)	8.14	7.2–63.4
Cortisol peak (ng/ml)	0.49	4.26–24.85
PRL (ng/ml)	26.6	3.6–16.3
FSH (mIU/ml)	<1.00	2.1–18.6
LH (mIU/ml)	<0.20	1.7–11.2
Estradiol (pg/ml)	<25.00	<75
Progesterone (ng/ml)	<0.10	<0.46
Testosterone (ng/dl)	3.12	262–870
Antibodies		
RF (KIU/L)	31.8	<14.0
AKA	Negative	Negative
CCP (RU/ml)	3.3	≤5.0
RA33 (AU/ml)	6.42	<25
ANA	Positive (1:1,000)	Negative
Anti-Sm	Positive (++)	Negative
Anti-SSA	Negative	Negative
Anti-SSB	Positive	Negative
Anti-dsDNA	Positive (+)	Negative
ACA	Negative	Negative
AHA	Negative	Negative
Anti-nucleosome	Negative	Negative
ARPA	Negative	Negative
Anti-Jo-1	Negative	Negative
Anti-nRNP	Positive (++)	Negative
Anti-Ro-52	Negative	Negative
Anti-Scl-70	Negative	Negative
ACL-IgG	Positive	Negative
LA1 (s)	27.10	31–44
LA2 (s)	30.5	30–38
LA1-LA2 (ratio)	0.89	1.0–1.2
Image		
Pituitary MRI	Empty sella	Normal
Adrenal MRI	Normal	Normal

HbA1c, glycated hemoglobin A1c; cTnI, cardiac troponin I; FT4, free T4; FT3, free T3; TSH, thyroid stimulating hormone; GH, growth hormone; IGF-1, insulin-like growth factor 1; ACTH, corticotrophin; PRL, prolactin; FSH, follicle-stimulating hormone; LH, luteinizing hormone; RF, rheumatoid factors; AKA, anti-keratin antibody; CCP, cyclic citrullinated peptide antibody; ANA, antinuclear antibodies; anti-SSA, anti- Sjögren’s syndrome A; anti-SSB, anti- Sjögren’s; syndrome B; anti-dsDNA, anti-double-stranded DNA antibodies; ACA, anti-centromere autoantibody; AHA, anti-histone antibody; ARPA, anti-ribosomal P protein antibodies; ACL-IgG, anticardiolipin antibody-IgG; LA1, lupus anticoagulant 1; LA2, lupus anticoagulant 2; MRI, magnetic resonance imaging.

Based on the above, the patient was diagnosed with AH, anterior pituitary hypofunction, SLE, NIDDM, and CVD. According to the clinical guidelines (6, 7), the patient was treated with 30 mg/d prednisone, 80 mg/d testosterone undecanoate, and 50 µg/d levothyroxine for the treatment of anterior pituitary hypofunction; 0.4 g/d hydroxychloroquine for the treatment of SLE; 22 U/d premixed human insulin for the treatment of NIDDM; and 75 mg/d clopidogrel, 10 mg/d atorvastatin calcium tablets, 50 mg/d captopril and 100 mg/d metoprolol for the treatment of CVD. Three days after the treatment, the sodium level had risen to 134 mmol/L, and the patient’s symptoms resolved gradually. Then, the patient was discharged with prescriptions for oral administration on the 11th hospital day. The dosage of prednisone was decreased after one month, with a monthly decrease of 5 mg until it was maintained at 10 mg/d. Two months after discharge, the patient reported no fatigue, anorexia, drowsiness, or leg oedema, and the patient’s sodium level was normal at the follow-up visit.

## Review of the Literature

We conducted a literature search with the terms “autoimmune hypophysitis” or “lymphocytic hypophysitis” and “systemic lupus erythematosus” in the PubMed, Embase, and CNKI databases for case reports of AH with SLE from inception to August 10, 2020. Then, manual searching was conducted according to the references of relevant acquired articles. Five cases of AH with SLE were reported (2, 8–11). Unfortunately, we did not obtain the complete publication of the first case reported by Hasegawa et al. (8). We summarized the remaining 4 cases in **Table 2** (2, 9–11).

**Table 2:** Reported cases of AH associated with SLE.

No.	Source	Age (years)/Gender	Diagnosis	Clinical presentation	Pituitary defects	Treatment	Glucocorticoid dose	Prognosis
1	Katano et al., (9)	26/Female	AH with SLE	Visual disturbances	Panhypopituitarism	Surgery	Not provide	Not provide
2	Ji et al. (2)	20/Female	AH with SLE	Headache and nausea	Anterior pituitary hypofunction	Trans-sphenoidal surgery and medication with prednisolone and hydroxychloroquine	Prednisolone 15 mg/d	Remission
3	Hashimoto et al. (10)	27/Male	AH with SLE	Polyuria and polydipsia, facial erythema and general malaise.	Panhypopituitarism	Medication with hydrocortisone and prednisolone.	Prednisolone 30 mg/d	Remission
4	Huang et al. (11)	19/Female	AH with SLE	Amenorrhea, polyuria and polydipsia	Normal	Medication with methylprednisolone, desmopressin, azathioprine, and hydroxychloroquine	Methylprednisolone: 200 mg/d*3d→160 mg/d*3d→ 100 mg/d*3d→80 mg/d*3d Prednisone: 40mg/d→30mg/d	Died

AH, autoimmune hypophysitis; SLE, systemic lupus erythematosus.

In the four cases of AH with SLE, only one patient was male, and the remaining patients were female. The onset age ranged from 19 to 27 years. Three of them were SLE patients who then developed visual impairment, headache, nausea, polyuria, or polydipsia. One patient was previously diagnosed with diabetes insipidus and then presented with facial erythema and general malaise. Most of the patients were diagnosed based on clinical and imaging features. Two patients underwent surgery, and postoperative pathological examination confirmed the diagnosis. Three patients were treated with glucocorticoid medication. One patient received hydroxychloroquine and azathioprine for the treatment of SLE. Three patients achieved remission, but one died of severe hyponatraemia.

## Discussion

AH is related to a disruption of tissue organization and frequently leads to glandular malfunction, which generally manifests as a mass within the sella turcica, with headache and/or visual disturbances (5). Pituitary enlargement is often secondary to infiltration and oedema. The progression of AH includes remission, spontaneous or pharmacological resolution of the inflammation, or progressive diffuse destruction with glandular atrophy for fibrotic replacement, thus resulting in varying degrees of pituitary dysfunction (1) and symptoms of hypopituitarism, including low ACTH, low TSH, low gonadotropins, or low prolactin (5). Currently, a definitive diagnosis of AH can be based only on pathological examination of a pituitary biopsy sample after invasive surgical intervention. For patients with glandular atrophy, as in the present case, clinicians should make a non-invasive diagnosis through clinical and imaging features and then treat with conservative management with glucocorticoids and other anti-inflammatory and immunosuppressive agents (methotrexate, azathioprine) (12), which aims to reduce inflammation and restore pituitary function.

SLE is a chronic autoimmune disease in which the immune system attacks healthy cells and tissues throughout the body, and the most frequently targeted organs are the kidneys, skin, lungs, brain, and heart (13). AH and SLE are both autoimmune diseases, but we are not aware of any histologically proven description or mechanism that links AH with SLE. It is still unclear whether the pituitary is a target of SLE. Our case demonstrates a potential association between AH and SLE. AH may need to be considered in the evaluation of SLE patients with headache, hyperprolactinemia, a pituitary mass, and hypopituitarism.

## Conclusion

In conclusion, we report the case of a 66-year-old man with AH and SLE who presented with hypopituitarism and hyponatraemia. The diagnosis was made mainly based on clinical manifestations, laboratory assessment, and imaging studies. The treatment approach, with glucocorticoids and immunosuppressive agents, resulted in long-term remission of symptoms of hypopituitarism and hyponatraemia.

## Data Availability Statement

The original contributions presented in the study are included in the article/supplementary material; further inquiries can be directed to the corresponding author.

## Ethics Statement

Written informed consent was obtained from the individual(s) for the publication of any potentially identifiable images or data included in this article.

## Author Contributions

HJZ and PX collected data and wrote the manuscript. QW, HZ, and CL provided suggestion during the diagnosis and treatment in this case. All authors contributed to the article and approved the submitted version.

## Conflict of Interest

The authors declare that the research was conducted in the absence of any commercial or financial relationships that could be construed as a potential conflict of interest.
